# Supplementary material for: PLEK2, RRM2, GCSH: A Novel WWOX-Dependent Biomarker Triad of Glioblastoma at the Crossroads of Cytoskeleton Reorganization and Metabolism Alterations
Source: Cancers (Basel). 2021 Jun 12;13(12):2955. doi: 10.3390/cancers13122955 (PMC8231639; doi:10.3390/cancers13122955)
Supplement: Supplementary file 1 [file cancers-13-02955-s001.zip › Table S1.pdf]

Table S1. Role of the top genes in glioma and cytoskeleton

Gene symbol	Importance in glioma	Participation in cytoskeleton regulation	Reference
BMP4	Strong independent prognostic factor; predicts metastasis and postoperative prognosis; inhibits GBM invasion via increase of *CDH1* and *CLDN1* expression	Re-organizes actin dynamics and activates Rac1, Rho, Cdc42	[105-107]
C15orf48 (also: *NMES1*)	One of the most upregulated genes but in human spinal cord reactive astrocytes; may be implicated in the cell cycle regulation	*None or not yet investigated*	[108]
CCL11	One-year survival predictor in GBM patients; upregulated in GBM, promotes proliferation, invasion and migration	Potentially drives cellular motility through actin cytoskeleton polymerization which is modulated by its receptor i.e. CCR3	[109, 110]
CMTM6	Diminishes T-lymphocyte-dependent anti-tumor immunity; Reduces survival and indicates poor prognosis	*None or not yet investigated*	[111]
COL3A1	Correlates with glioma grade; potential diagnostic and therapeutic biomarker; increases proliferation and migration	*None or not yet investigated*	[112, 113]
CUX2 (also: *CUTL2*)	Its homolog, *CUX1*, correlates with poor prognosis in gliomas; on its own, *CUX2* is considered an oncogene in thyroid cancer as it induces EMT, and influences AKT or mTOR phosphorylation	Its loss leads to the reduced β-actin expression which is important for synapses stabilization and dendrite branching	[114, 115]

DUSP7	Downregulated in GBM	Guides chromosomes alignment; chromosome dynamics is known to be regulated by cytoskeletal proteins	[116-118]
FAM92B (also: *CIBAR2*)	Proposed as risk factor of gliomas during survival prediction modeling	As one of BAR domain-containing proteins, regulates actin cytoskeleton	[119, 120]
GCSH	"Switch gene" (implicated in drastic cell phenotype changes) in GBM	*None or not yet investigated*	[121]
GLB1 (also: *EBP*)	Allows glioblastoma to adhere to matrix, increasing aggressiveness	*None or not yet investigated*	[122]
GRIN2B	Allows glutamate-dependent synaptic transmission which is frequently dysfunctional in gliomas; ion channel enriched in stem-like cells of GBM	Interacts with cytoskeletal protein α-actinin-2 via carboxyl-terminal domain; α-actinin-2 is closely associated with multimerins which are possible markers and therapeutic targets in LGG	[123-126]
HOXA1	Upregulated in GBM, inversely correlates with survival of glioma patients	CDH1-dependent signaling increases *HOXA1* expression through Rac1; the same signaling regulates actin cytoskeleton at cadherin adhesive contacts	[127, 128]
HOXA10	Regulates homologous recombinant DNA repair hence temozolomide resistance in GBM; promotes tumorigenesis in glioma	Alterations in HOXA10-dependent *CK15* gene expression facilitate cytoskeleton remodeling	[129-131]

KIF20A	Indicates poor prognosis of glioma patients; downregulation leads to apoptosis and cell cycle arrest via PI3K/AKT suppression	Interacts with Rab6 to regulate Golgi-related vesicles trafficking; also essential for cytokinesis	[132]
LBP	Upregulated in high-grade glioma; potential serum diagnostic marker of treatment response	*None or not yet investigated*	[133]
MMP13	Increases GBM migration and invasion; abundantly expressed in GBM stem-like cells; poor survival when overexpressed	*None or not yet investigated*	[134, 135]
MTHFD2	Correlates with glioma tumor grade and poor prognosis	Its depletion leads to vimentin organization defects	[72, 136]
NF2	Possesses oncogenic properties when phosphorylated at S518 in GBM; S518 phosphorylation inactivates tumor suppressor capabilities and affects EGFR or Notch1 and downstream targets *HES1* or *CCND1*	Relates to ezrin, radixin, moesin i.e. critical members allowing anchorage between membrane proteins and cortical cytoskeleton; reverses the actin-dependent cytoskeletal phenotype	[137-139]
PHF5A	Its knockdown results in reduced GBM viability and cell cycle arrest	*None or not yet investigated*	[140]
PLEK2	Overexpressed in GBM and might contribute to oxidative phosphorylation; promotes invasion and metastasis via EGFR/CCL2 signaling but in gallbladder cancer	Required for actin cytoskeleton integrity	[81, 141, 142]
RNF141 (also: *ZNF230*)	Member of genes network predicting GBM prognosis	*None or not yet investigated*	[143]

RPS27	Correlates with age in IDH-mutated glioma patients and with Ki67 in GBM patients; detected in astrocytic tumors but not normal astrocytes	*None or not yet investigated*	[144]
RRM2	Promotes GBM tumorigenicity and protects from endogenous replication stress through the BRCA1-RRM2 axis; regulates glioma proliferation and migration via ERK1/2 and AKT signaling	Its downregulation decreases hPLIC1 which is implicated in actin cytoskeleton re-organization	[55, 145-147]
RXRG	May contribute to antineoplastic effect when bound with ligands i.e. retinoids	*None or not yet investigated*	[148]
SAA2	Increases GBM proliferation and invasion	*None or not yet investigated*	[149]
SPOCK1	Contributes to GBM metastasis and resistance to temozolomide; promotes glioma invasion, migration, proliferation via Wnt/β-catenin and PI3K/AKT pathways	Its upregulation correlates with cortical cytoskeleton EPB41L4B protein	[150-152]
TAF10	Presumably important for proliferation of undifferentiated carcinoma cells or for efficient transcription (based on data from embryonal carcinoma or breast cancer, respectively)	*None or not yet investigated*	[153]
TTR	Overexpressed in high-grade gliomas compared to low-grade or normal brain tissue	Interacts with RAGE receptor which is implicated in actin cytoskeleton remodeling	[154-156]

UHRF1	Decreases p16^{INK4A}, promoting GBM proliferation	Contributes to microtubule cytoskeleton organization through its downstream targets: *BRCA2*, *HOOK1*, *KIF11*, *KIF18A*	[157, 158]
WT1	Increases proliferation and decreases apoptosis of GBM	Interacts with actin both in cytoplasm and nucleus; supposedly binds to RNA on cytoskeleton-dependent regulation manner	[159, 160]
